# Epidemiology of cognitive impairment in rural and urban settings in southern Peru

**DOI:** 10.1002/alz.092130

**Published:** 2025-01-09

**Authors:** Claudia Rivera‐Fernandez, Eder Herrera‐Perez, Marcio F. Soto‐Añari

**Affiliations:** ^1^ Laboratorio de Neurociencia, Universidad Católica San Pablo, Arequipa Peru; ^2^ Instituto Peruano de Neurociencias, Lima Peru; ^3^ Grupo de investigación Molident, Universidad San Ignacio de Loyola, Lima Peru; ^4^ Universidad Católica San Pablo, Arequipa Peru

## Abstract

**Background:**

In Peru, the prevalence of dementia is associated with a significant number of social, ethnic, and demographic factors. Data on prevalence is still limited, especially in regions distant from major urban centers. Our objective was analyze the prevalence of dementia in the southern population of Peru.

**Method:**

We conducted an epidemiological study on cognitive impairment and dementia in southern Peruvian populations. A total of 314 older adults (mean age = 68.45, SD = 9.57), comprising 74.03% women and 25.96% men, were recruited from the city of Arequipa in the southern region of Peru. We applied a clinical protocol that included brief cognitive tests (Addenbrooke Cognitive Examination and Rowland Universal Dementia Assessment Scale (RUDAS)). Functional status measures were evaluated using the Pfeffer questionnaire (PFAQ). Additionally, a brief questionnaire on social determinants of health and clinical health information was administered. The cut‐off point of the brief cognitive test and functional measurement was applied to approximate the diagnosis. Binary logistic regression was performed with positive and negative cases as the dependent variable and gender, education, and ethnicity as predictors.

**Result:**

We observed a global prevalence of 7.35%, which can vary according to the number of years of education. Individuals with more than 6 years of education showed a prevalence of 10.71%, while low‐educated populations exhibited a higher prevalence (23.53%). Additionally, we observed some minor variations based on ethnicity, health issues, and emotional states.

**Conclusion:**

We identified a lower prevalence of cognitive impairment compared to previous reports. Social determinants of health appear to exert a significant impact on prevalence. This initial study in the southern Peruvian population highlights a noteworthy interaction between nature and nurture in the risk of dementia.

